# Optimizing treatment of compulsive hair pulling in children and young people: A case series from a pediatric psychodermatology service

**DOI:** 10.1111/1346-8138.17136

**Published:** 2024-03-06

**Authors:** Brent J. Doolan, Rukshana Ali, Susannah E. Baron

**Affiliations:** ^1^ Guy's and St. Thomas' NHS Foundation Trust London UK; ^2^ St. John's Institute of Dermatology, School of Basic and Medical Biosciences King's College London London UK

**Keywords:** : compulsive hair pulling, pediatrics, psychodermatology, trichotillomania

Compulsive hair pulling, is an often‐debilitating disorder characterized by repetitive pulling out of one's own hair, leading to hair loss that is not caused by another medical condition, but is often triggered by psychological distress.^1^ Pediatric epidemiologic studies are lacking, but the condition has an estimated prevalence of 1%–3%.^2^ It is most common in early adolescence, but can develop at any age group and affect any hair‐bearing region. Patients report difficulty in controlling hair pulling, which often results in shame, embarrassment, and social avoidance.^1^ Due to cosmetic concern and the patchy loss of hair, which can resemble alopecia areata, pediatric patients are often referred into specialty dermatology clinics for management.^3^

In 2019, a multidisciplinary psychodermatology service was established for children and young people at Guy’s and St. Thomas’ NHS Foundation Trust, London. This comprised a pediatric clinical psychologist and consultant dermatologist accepting primary, secondary, and tertiary referrals. Prior to COVID‐19, a face‐to‐face model of care was implemented, whilst during COVID‐19 restrictions this model was changed to a virtual model and subsequently became a hybrid/mixed model after COVID‐19 restrictions were eased (Supporting Information Figure S1). Patients and their families attended 45–60‐min sessions every 2–3 months, with additional psychology sessions depending on clinical need. All patients were initially reviewed face‐to‐face for a scalp examination and trichoscopy, and reviewed in‐person prior to discharge.

A retrospective review of patients referred for compulsive hair pulling between February 2019 and July 2022 was undertaken. Eleven patients (9/11 [81.8%] female, mean 11.1 ± 3.6 years) were diagnosed with compulsive hair pulling. Most patients preferred a combination of face‐to‐face and virtual psychodermatology sessions (Supporting Information Table S1). Four patients were found to have an additional diagnosis of alopecia areata. We noted 5/11 (45.4%) patients had complete resolution of hair pulling, whilst 4/11 (36.4%) patients had a substantial reduction and 2/11 (18.2%) now have stable disease. The mean number of psychodermatology appointments required for a significant reduction or resolution of hair pulling was three sessions (paired *t*‐test, *P* < 0.01). We noted multiple emerging themes as contributing to compulsive hair pulling (Figure 1).

**FIGURE 1:**
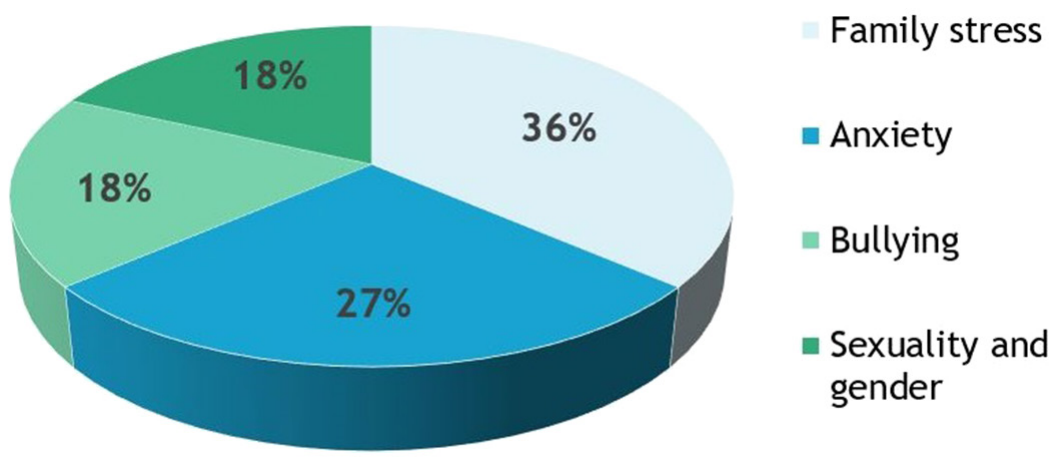
Emerging themes identified during psychodermatology sessions in pediatric patients with compulsive hair pulling.

The psychodermatology sessions were based on an assessment of the patient using the antecedent‐behavior‐consequence model drawn from cognitive behavioral therapy, which aims to examine behaviors patients want to change, the triggers behind those behaviors (antecedents), and the impact of those behaviors on maladaptive patterns (consequences).^4^, ^5^ Based on this assessment, individualized models of care were implemented. These models included family therapy (the patients’ interactions in their social world), cognitive behavioral therapy (mindfulness, relaxation, and habit reversal training), and acceptance commitment therapy (what is important to each young person). For example, one patient was found to pull her hair when she was alone in her bedroom. We implemented a family model of care, where she would sit out with her family to complete homework instead of alone in her room. She also enjoyed seeing the roots of the hairs she pulled out, so this behavior was replaced with a similar manual activity of pulling stems from a cress plant, which allowed her to examine the roots and gave her the same sense of satisfaction experienced with hair pulling.

On review of the emerging themes, we found that, especially with girls, parents encouraged them to have long hair, but they either identified as male and/or knew they had a preference to the same sex. This would lead to “jokes” about being gay, which caused shame and further exacerbated the ongoing cycle of distress. Surprisingly, the two patients who had gender/sexuality issues disclosed this information during virtual sessions, indicating that they felt safe within our clinic and this permitted psychology‐facilitated discussions with parents around these topics.

Unlike previous published psychodermatology services,^6^ COVID‐19 has provided an avenue to explore hybrid models of care, which may facilitate engagement as young people are able to express themselves whilst in the safety of their own bedrooms, rather than attending a hospital appointment. We also noted that a proportion of patients had a dual diagnosis of hair pulling and alopecia areata, which emphasizes the importance of examination and trichoscopy, as well as a thorough psychosocial history.^3^ However, it must be recognized that repeated acts of hair extraction in the same area can lead to permanent hair loss even when treatment for hair pulling is successful, and this should be communicated to the patient/family when reviewed. This case series highlights the importance of a multidisciplinary team approach to the care of children and young people with compulsive hair pulling and emphasizes the support and safe space needed to promote engagement with psychological therapy.

## FUNDING INFORMATION

No funding sources to declare.

## CONFLICT OF INTEREST STATEMENT

All authors of this manuscript certify that they have no affiliations with or involvement in any organization of entity with any financial interest or other equity interest or non‐financial interest in the materials discussed in this manuscript.

## Supporting information


Supporting Information Data S1.
