# Supplementary material for: Optimizing treatment of compulsive hair pulling in children and young people: A case series from a pediatric psychodermatology service
Source: J Dermatol. 2024 Mar 6;51(4):612–3. doi: 10.1111/1346-8138.17136 (PMC11484137; doi:10.1111/1346-8138.17136)
Supplement: Supplementary file 1 — Supporting Information Data S1. [file JDE-51--s001.docx]

**SUPPLEMENTARY FILE:**

**
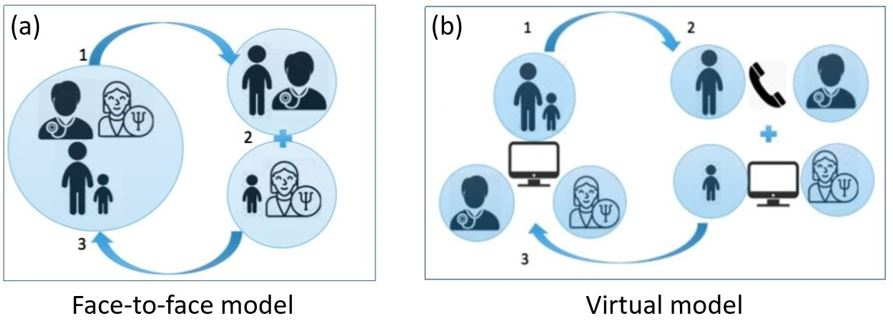
**

**Figure S1:** An overview of the multidisciplinary paediatric psychodermatology service at Guy’s and St. Thomas’ Hospital NHS Trust. **(a)** Prior to COVID-19 restrictions, a face-to-face model was undertaken, where the family are seen together with the clinical psychologist and dermatologist to assess the skin issue and any psychological issues. They then split apart, where the patient stays with the psychologist and the parent(s) leave to discuss the issue with the parent(s). All parties then meet once again and discuss the issues identified and the plan. **(b)** During COVID-19 restrictions, the face-to-face model changed to a virtual model, where all parties meet on a video platform. The patient then stays on the video platform with the psychologist, whilst the parent(s) are called on the telephone by the dermatologist. All parties then meet on the video platform and discuss the issues identified and the plan.

**Table S1:** An overview of children and young people undergoing treatment for compulsive hair pulling at the multidisciplinary paediatric psychodermatology service at Guy’s and St. Thomas’ NHS Foundation Trust, London between February 2019 to July 2022.

| **Case** | **Age** | **Sex** | **Place of residence** | **Referrer** | **Additional issues** | **Psychodermatology appointments** | **1:1 Psychology appointments** | **Format*** | **Outcome** |
| --- | --- | --- | --- | --- | --- | --- | --- | --- | --- |
| 1 | 13 | F | Greater London | Primary | Alopecia areata | 4 | 2 | Virtual | Reduction |
| 2 | 11 | F | City of London | Primary | Alopecia areata | 2 | 1 | Face-to-face | Resolution |
| 3 | 12 | F | Greater London | Primary | - | 2 | 0 | Virtual | Resolution |
| 4 | 9 | F | Greater London | Secondary | - | 4 | 1 | Hybrid | Reduction |
| 5 | 15 | F | Greater London | Tertiary | - | 4 | 1 | Hybrid | Resolution |
| 6 | 10 | M | Greater London | Primary | Atopic dermatitis | 3 | 0 | Hybrid | Resolution |
| 7 | 14 | F | Regional England | Tertiary | - | 4 | 2 | Virtual | Reduction |
| 8 | 11 | M | City of London | Primary | Alopecia areata | 3 | 1 | Face-to-face | Resolution |
| 9 | 10 | F | Greater London | Primary | Atopic dermatitis | 2 | 0 | Hybrid | Stable |
| 10 | 12 | F | Regional England | Secondary | Skin picking | 2 | 1 | Virtual | Reduction |
| 11 | 15 | F | Greater London | Tertiary | - | 1 | 2 | Hybrid | Stable |
| *Please note that all patients had an initial consultation that was face-to-face, which included a full scalp examination and trichoscopy to allow diagnosis. | | | | | | | | | |
